# Relationship of lower uterine segment cancer with Lynch syndrome: A novel case with an *hMLH1* germline mutation

**DOI:** 10.3892/or.2012.2008

**Published:** 2012-08-31

**Authors:** KENTA MASUDA, KOUJI BANNO, AKIRA HIRASAWA, MEGUMI YANOKURA, KOSUKE TSUJI, YUSUKE KOBAYASHI, IORI KISU, ARISA UEKI, HIROYUKI NOMURA, EIICHIRO TOMINAGA, NOBUYUKI SUSUMU, DAISUKE AOKI

**Affiliations:** Department of Obstetrics and Gynecology, School of Medicine, Keio University, Tokyo 160-8582, Japan

**Keywords:** endometrial cancer, lower uterine segment, Lynch syndrome, *hMLH1*

## Abstract

Lynch syndrome is a genetic disease that often develops in patients with endometrial cancer and is caused by abnormal DNA mismatch repair (MMR) genes. In the United States, it was recently reported that the prevalence of Lynch syndrome with an *hMSH2* mutation in patients with endometrial cancer in the lower uterine segment (LUS) is much greater than that in patients with endometrial cancer, although no such reports have been published in Asia. In this study, we examined the correlation between endometrial cancer in LUS and abnormalities in MMR genes. We examined 625 patients, who were diagnosed with endometrial cancer and underwent a hysterectomy. Nine patients (1.4%) had cancer based on pathological confirmation of a tumor in the lower part of the uterus and no cancer in the upper part. These cases were compared with 27 cases of sporadic endometrial (non-LUS) cancer. The age and BMI of the patients with LUS cancer were significantly lower than those of the patients with non-LUS cancer. No differences were observed in the pathological characteristics. The microsatellite instability (MSI)-positive rates were similar. Immunohistochemistry showed a decreased expression of *hMLH1* and *hMSH6* in patients with LUS cancer. In contrast with earlier reports from the United States, hMSH2 was expressed in all the cases. Of the 2 patients with LUS cancer who exhibited high MSI, 1 patient showed abnormal methylation of *hMLH1*, while the other patient was diagnosed with Lynch syndrome with a mutation in the *hMLH1* gene. This is the second report on the relationship of LUS cancer and Lynch syndrome, and the first to describe an Asian patient with LUS cancer with Lynch syndrome induced by an *hMLH1* mutation.

## Introduction

There were 47,130 cases of endometrial cancer in the United States in 2012 with fatalities of 8,010. Endometrial cancer accounts for approximately 6% of all cancers in women (1). The incidence of cervical cancer has been less frequent in developed countries, as a result of check-ups and vaccine development (2), the incidence of endometrial cancer, however, is increasing in these countries (1,3). Most causes of endometrial cancer are associated with the effect of estrogen on the endometrium, while unopposed estrogen in association with obesity is also a major risk factor (3,4).

Approximately 5% of the endometrial cancer cases are thought to result from genetic predisposition (5). One of the most important genetic diseases, Lynch syndrome [hereditary non-polyposis colorectal cancer (HNPCC)], is a hereditary syndrome associated with familial cancers, including colorectal cancer and Lynch syndrome-related cancers, such as endometrial cancer (6). The cause of the disease is the germline mutation of DNA mismatch repair (MMR) genes *MLH1, MSH2, MSH6* and *PMS2*, characterized by autosomal dominant inheritance (7). The risk for cancer development in Lynch syndrome depends on the cancer type and is particularly high in colorectal and endometrial cancers. Thus, in patients with Lynch syndrome the risk of developing colorectal cancer throughout life is approximately 80% and 40–60% in males and females, respectively, while that of developing endometrial cancer throughout life is 40–60%. The risks of developing gastric, ovarian, small intestine, renal pelvic, ureteral, brain and biliary tract cancers are 13, 12, ≤5, 4, 4 and 2%, respectively (8,9).

Methods to identify Lynch syndrome-related endometrial cancer are important for several reasons. First, endometrial cancer is likely to be the primary cancer in female individuals with Lynch syndrome and may therefore be managed by prevention and screening. Endometrial cancer develops first in 51% of females with Lynch syndrome, followed by colorectal cancer at an average of 11 years after the onset of endometrial cancer. Thus, endometrial cancer frequently occurs as a sentinel cancer, with its identification likely to be useful for the prevention of the secondary cancer (10). A secondary reason requiring the identification of useful methods is that the diagnosis of Lynch syndrome provides a basis for the examination of gene mutations in family members. In the case of an identical mutation, appropriate genetic counseling, screening and preventive therapy are offered to family members.

Examination of the familial history of cancer is particularly useful for identifying Lynch syndrome-related endometrial cancer. The Amsterdam II criteria for Lynch syndrome are based on familial history and age (11). These criteria were developed from the original Amsterdam criteria, which included only familial history for colorectal cancer (12). However, certain patients in small families may not meet the diagnostic criteria, while others with *hMSH6* mutation do not meet the Amsterdam II criteria due to having developed cancers at an older age (13,14). Lynch syndrome in women is associated with a relatively early onset of endometrial cancer (15,16) as well as the development of a secondary cancer as a double cancer (17). These characteristics should lead to suspicion of Lynch syndrome.

Microsatellite instability (MSI) reflects abnormality in DNA MMR (18). Microsatellites are short DNA repeat sequences that increase or decrease in number when MMR is dysfunctional. A MSI test is recommended before examining germline mutation in patients with suspected Lynch syndrome. MSI is detected in Lynch syndrome caused by germline mutation and in sporadic endometrial cancer caused by epigenetic aberrant methylation in the promoter region of *hMLH1*(19). However, MSI in these two diseases is distinguished based on the detection of MMR protein levels, using a combination of immunohistochemistry (IHC) and detection of hypermethylation in the *hMLH1* promoter region (20,21). The Bethesda criteria determine the situation in which a MSI test should be conducted for colorectal cancer (22). Similar guidelines have been developed for endometrial cancer (23), although not including items regarding histopathological findings, in comparison with the Bethesda criteria.

Tumors in LUS have been found to be frequently associated with Lynch syndrome (24). The endometrium comprises the regions: the uterine corpus (UC) and the LUS (25). Endometrial cancer usually develops in the mucosa of the UC and the uterine fundus, but only occasionally in the LUS. When a tumor is macroscopically observed to develop in LUS and expand from the lower UC to the upper cervix, the disease is defined as LUS cancer (26). Pathologically, LUS cancer is a poorly differentiated (G3) adenosquamous carcinoma, although the tumor size is small (26,27–29). Westin *et al* first showed a relationship between LUS cancer and Lynch syndrome in 35 patients with cancer in the uterine isthmus (24). Decreases were found in the MSH2 and MSH6 protein levels, using IHC in 10 patients (29%), including 4 cases with strongly suspected *hMSH2* mutations due to high MSI and 1 case with a decreased MLH1 protein level with no aberrant methylation. These 5 patients (14.2%) met the Amsterdam II criteria and were diagnosed with Lynch syndrome, having *hMSH2* mutations, MSI and a decreased MSH2 protein level in IHC (24).

In the case that LUS cancer is found to occur frequently in patients with Lynch syndrome in additional large-scale studies, it might be added to the clinical characteristics used to identify Lynch-related endometrial cancer. Therefore, we conducted the first study on the relationship between the clinicopathological characteristics of LUS cancer and Lynch syndrome in Asian patients.

## Materials and methods

### Case selection

The subjects were patients diagnosed with endometrial cancer, who underwent hysterectomy in our hospital between January, 2002 and July, 2010. Based on pathology reports, patients were divided into LUS and non-LUS groups. The criterion for the diagnosis of LUS cancer was the macroscopic observation of a tumor developing in the LUS and expanding from the lower uterine corpus to the upper cervix. Patients with tumors in the LUS or in any other region and those with cancer spreading from the fundus to the endocervix, for whom the site of cancer onset was unclear were excluded from the study.

Clinical data were collected from patient records. Surgical staging was determined based on the criteria of the 1988 International Federation of Gynecology and Obstetrics (FIGO) Classification or the Guidelines for Endometrial Cancer published by the Japan Society of Obstetrics and Gynecology. Pathological evaluation was performed with hematoxylin and eosin staining. Lynch syndrome was diagnosed using the Amsterdam II criteria. This study was conducted after the approval of the institutional review board.

### Immunohistochemistry

**Figure 1:**
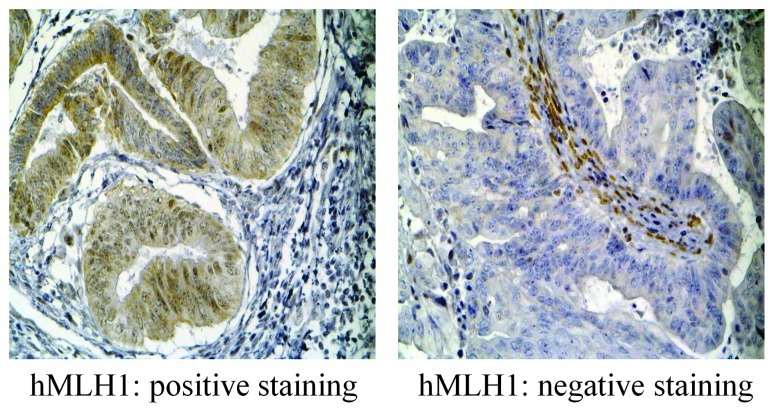
Left endometrial cancer shows positive staining of the nuclear expression of hMLH1. Right endometrial cancer shows loss of the nuclear expression of hMLH1.

Immunohistochemical staining was performed on 2-μm sections of formalin-fixed, paraffin-embedded tissues using standard procedures. Slides were cleaned in xylene and dehydrated in graded alcohol. Antigen retrieval was performed with a 10-min microwave treatment in 10 mM citrate buffer, pH 7.0. Endogenous peroxidase was blocked by dipping sections in 0.3% H_2_O_2_ in methanol for 10 min. Slides were incubated with mouse monoclonal antibody to hMLH1 (clone G168-15; Pharmingen, San Diego, CA, USA) (1:30), mouse monoclonal antibody to hMSH6 (clone 44; BD Transduction Laboratories, San Jose, CA, USA) (1:500), or rabbit polyclonal antibody to hMSH2 (SC-494; Santa Cruz Biotechnology, Santa Cruz, CA, USA) (1:200) for 90 min at room temperature. Immunostaining was performed using the avidin-biotin-peroxidase complex method with an Elite ABC kit (Vector Laboratories, Burlingame, CA, USA), using 3,3′-diaminobenzidine as a chromogen and H_2_O_2_. Slides were counterstained with hematoxylin, dehydrated in graded alcohol, dried and coverslipped. A normal nuclear staining pattern was detected for hMLH1, hMSH2 and hMSH6, while nuclei in the stromal cells were used as internal positive controls (Fig. 1).

### MSI analysis

DNA was extracted from paraffin-embedded tumor tissue and normal tissue using DEXPAT (Takara, Shiga, Japan) for use in polymerase chain reaction (PCR) assays. DNA samples were analyzed using five microsatellite primers, recommended by the National Cancer Institute (NCI), which amplified D2S123, D5S376, D17S250, BAT25 and BAT26. The PCR primers for these regions were purchased from Research Genetics (Huntsville, AL, USA). The antisense primers contained a fluorescent marker, Cy5 amidite (indodicarbocyanine), at their 5′ ends. AmpliTaq polymerase and AmpliTaq buffer (Perkin-Elmer, Boston, MA, USA) were used in the PCR, in which 1 μl of sample DNA (template, 0.1 μg/μl) was added to 24 μl of premixture (distilled water, 16.125 μl; 1.25 mmol/l dNTP, 4 μl; 10× BPCR buffer, 2.5 μl; optical density (OD) 2.2 forward primer, 0.625 μl; OD 2.2 reverse primer, 0.625 μl; and *Taq* polymerase, 0.125 μl) in a total reaction volume of 25 μl. Forward and reverse primers specific for the D2S123, D5S376, D17S250, BAT25 and BAT26 regions were used. DNA denaturation at 95°C for 5 min was followed by 40 cycles for 30 sec at 95°C, 40 sec at 55°C and 40 sec at 72°C. The reaction mixture was then heated to 72°C for 7 min, cooled and stored at 4°C. The PCR products were combined with size markers, denatured for 5 min at 80°C, and then electrophoresed in 6% Long Ranger 7 M urea denaturing gel on an AFL red DNA sequencer (Amersham Pharmacia Biotech, Tokyo, Japan). DNA fragment sizes were analyzed using gene scanning software (Allele Links; Amersham Pharmacia Biotech). Tumors showing an allelic shift at ≥2 markers were classified as MSI-H, while tumors with an allelic shift at 1 marker were classified as MSI-L, and those with no allelic shift at any marker as microsatellite stable (MSS).

### Methylation-specific PCR (MSP)

DNA (1 μg) extracted from paraffin-embedded tumor tissue was diluted with 50 μl of distilled water and incubated in 5.5 μl of 3 N NaOH at 37°C for 15 min. To this solution, 30 μl of 10 mM hydroquinone (Sigma, St. Louis, MO, USA) and 520 μl of 3 M sodium bisulfite (prepared at pH 5.5 with 10 N NaOH, Sigma) were added with mixing. Mineral oil was laid over the solution to prevent evaporation and the solution was incubated overnight at 50°C. The lower layer of the solution was then mixed with 1 ml of Clean-up Resin (Promega, Madison, WI, USA) and injected into a column. After rinsing with 2 ml of 80% isopropanol, the mixture was centrifuged at 15,000 rpm for 3 min to completely remove isopropanol. Then, 50 μl of hot (70°C) distilled water was added and the mixture was centrifuged at 15,000 rpm for 2 min to elute DNA. The DNA was then incubated with 5.5 μl of 2 N NaOH at 37°C for 20 min. Then, 66 μl of 5 N ammonium acetate and 243 μl of 95% ethanol were added and the mixture was incubated at −80°C for 1 h and centrifuged at 15,000 rpm for 30 min to precipitate DNA. The supernatant (>50 μl) was removed and 1 ml of 60% ethanol was added. The mixture was centrifuged at 15,000 rpm for 30 min and rinsed. The precipitated DNA was dried in air and dissolved in 20 μl of distilled water. The DNA solution (2 μl) was used as the MSP template. In the PCR assay, AmpliTaq Gold & 10× PCR buffer/MgCl_2_ with dNTP (Applied Biosystems, Foster City, CA, USA) were used and the results were analyzed with a GeneAmp PCR System 9700 (Applied Biosystems). The primer sequences were 5′-ACG TAG ACG TTT TAT TAG GGT CGC-3′ (sense) and 5′-CCT CAT CGT AAC TAC CCG CG-3′ (antisense), 159 bp. Primer sequences for the unmethylated reaction were 5′-TTT TGA TGT AGA TGT TTT ATT AGG GTT GT-3′ (sense) and 5′-ACC ACC TCA TCA TAA CTA CCC ACA-3′ (antisense), 165 bp. PCR was performed for 35 cycles (94, 60 and 72°C, each for 30 sec).

### Mutation detection

A mutation in *hMLH1* was detected in case 9, which was MSI-H and negative for DNA hypermethylation in the *hMLH1* promoter. Peripheral leukocyte DNA was extracted and the *hMLH1* mutation was analyzed by direct sequencing with primers, as previously described (13). DNA yielding altered bands was automatically sequenced and the result compared with the normal DNA sequence.

### Statistical analysis

Statistical analysis was performed using the IBM SPSS Statistics 19 software. Clinical and pathological variables were compared in the two groups using χ^2^ and Student's t-test. P<0.05 was considered to indicate a statistically significant difference.

## Results

### Clinical background

The subjects were 625 patients diagnosed with endometrial cancer, who underwent hysterectomy in our hospital between January, 2002 and July, 2010. Pathological reports were evaluated and 9 (1.4%) patients were diagnosed in the LUS group. Table I shows the clinical background of these 9 patients and of an additional 27, randomly selected from the non-LUS group. The patients in the LUS group were significantly younger (44.4 vs. 59.5 years old, P=0.001) and had a significantly lower BMI (18.5 vs. 22.7 kg/m^2^, P=0.002). Cesarean section had been performed in 2 patients (22.2%) in the LUS group and in 1 patient (3.7%) in the non-LUS group, with no statistically significant difference between these groups. There were also no statistically significant differences in the frequency of delivery or in the rates of infertility and diabetes between these groups.

### Pathological characteristics

A comparison of the pathological characteristics of the LUS and non-LUS cases is shown in Table II. The histological type was endometrioid in 8 (88.8%) and adenosquamous in 1 (11.1%) of the LUS cases. There were no statistically significant differences in histology, grade, stage, rates of vascular invasion and lymph node metastasis or the depth of myometrial invasion between the 2 groups.

### Microsatellite status and IHC characteristics

A comparison of the microsatellite status and IHC findings for the LUS and non-LUS groups are shown in Table III. The microsatellite status was MSI-H in 2 LUS cases (22.2%) and MSI-L in 1 (11.1%), and MSI-H in 7 non-LUS cases (25.9%). Loss of expression of hMLH1 was detected in 4 LUS cases (44.4%) and 4 non-LUS cases (14.8%). Loss of expression of hMSH2 was not detected in LUS patients, although was found in 4 non-LUS cases (14.8%). Loss of expression of hMLH6 was detected in 1 LUS case (11.1%) and 11 non-LUS cases (40.7%). No statistically significant differences were found in the microsatellite status and IHC findings between the 2 groups.

### Lynch syndrome and LUS cancer

**Figure 2:**
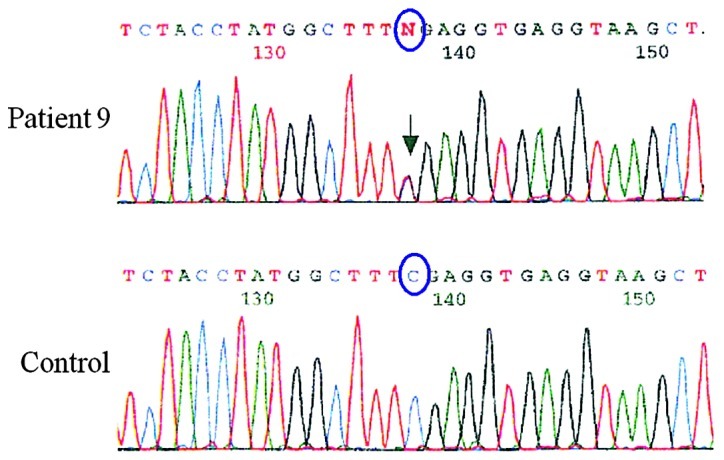
Germline mutation analysis of hMLH1. A nonsense mutation was identified at codon 100 in exon 3.

The microsatellite status, IHC findings for MMR genes, status of DNA hypermethylation of the *hMLH1* promoter and the Amsterdam II criteria for the 9 LUS patients are shown in Table IV. These 9 patients had hMSH2 expression in IHC. Cases 2 and 8 had a decreased hMLH1 protein level and were MSI-H and MSI-L, respectively. Hypermethylation of the *hMLH1* promoter was detected in the two patients. Case 9 had decreased hMLH1 in IHC, while the DNA methylation of the *hMLH1* promoter was not detected, despite the MSI-H status. Since the patient had a family history of cancer and met the Amsterdam II criteria, she was diagnosed with Lynch syndrome. Germ cell mutation of *hMLH1* was examined using peripheral blood lymphocytes and mutation from CGA to TGA was detected in codon 100 in exon 3 (Fig. 2).

## Discussion

In the present study, the incidence of LUS was 9 (1.4%) of the 625 patients with endometrial cancer, who underwent hysterectomy. This incidence is lower than the 3–6.3% rates of LUS cancer among the cases of endometrial cancer in previous studies (24,26,27). The patients with LUS cancer in this study were significantly younger than those with non-LUS endometrial cancer. At present, the largest study conducted comprised 35 patients with LUS cancer and 79 with non-LUS endometrial cancer. The mean onset ages were 54.2 and 62.9 years, respectively, indicating younger patients in the LUS group (24). In a study of patients with endometrial cancer aged ≤50 years, LUS cancer was present in 18% (16/88) (28). However, other small-scale studies have not shown similar results (26).

Endometrial cancer is classified into types I and II, with type I being estrogen-dependent. Obesity increases insulin resistance as well as the risk of endometrial cancer, due to an elevated blood estradiol level. Thus, BMI ≥25 doubles, while ≥30 triples the risk of endometrial cancer (30). Nulliparity, amenorrhea and infertility cause long-term stimulation by estrogen and are considered to be risks for endometrial cancer. In this study, BMI in the LUS group was significantly lower than that in the non-LUS group. The lower rate of obesity suggests that LUS cancer does not have the typical properties of type I endometrial cancer. Hachisuga *et al* found that the incidence of menstrual irregularity, nulliparity, infertility and polycystic ovary syndrome was significantly lower in the 16 patients with LUS cancer, compared with 72 patients with non-LUS endometrial cancer, suggesting that LUS cancer is not a typical type I endometrial cancer (28). In the present study, no difference was evident in the incidence of diabetes and infertility between the LUS and non-LUS groups.

Previous pathological findings demonstrated that a histological adenosquamous carcinoma, a higher grade (26–28) and muscle invasion (24,27,28,31) are common in LUS cancer. However, there was no significant difference in the pathological findings between LUS and non-LUS endometrial cancer in our patients.

The MSI-positive frequency in this study was 22.2% in the LUS and 25.9% in the non-LUS groups, with no statistically significant difference between the groups. Similar MSI-positive frequencies of 29% (127/441) and 21.7% (118/543) have been reported in previous studies that mainly included cases with hypermethylation of the *hMLH1* promoter (20,21).

In this study, hMLH2 expression in IHC was detected in all the patients with LUS cancer. These findings differ considerably from those of Westin *et al* who demonstrated a decreased hMSH2 and hMSH6 expression in 25.7% (9/35) of the cases (24). We found a decreased hMLH1 expression in 44.4% (4/9) of our cases of LUS cancer, with epigenetic suppression due to DNA hypermethylation of the *hMLH1* promoter detected in 2 cases (22.2%). This finding suggests that DNA hypermethylation of *hMLH1* also induces LUS cancer. A decreased hMSH6 expression was present in 11.1% (1/9) of the cases, while case 2 also had MSI-H. The MSI-positive rate associated with a reduced *hMSH6* expression is usually lower than that for *hMLH1* and *hMSH2*, while *hMSH6* knockout mice have been shown to have a negative MSI (32). However, the effect of a reduced *hMSH6* expression on MSI is unclear since case 2 also showed hypermethylation of *hMLH1*.

The incidence of Lynch syndrome in the LUS group was 11.1% (1/9) in this study, which is higher than the previously reported incidences of 1–2% for Lynch syndrome in patients with various types of endometrial cancer (20,21,33). Thus, the current small-scale study is the first to show a relationship between LUS cancer and Lynch syndrome in Asian patients. In their study, Westin *et al* found that the *hMSH2* mutation was causative in all the LUS cancer patients with Lynch syndrome (24); however, the *hMLH1* mutation was found to be causative in our study. This finding is the first evidence that LUS cancer is likely to develop due to the germline mutation of *hMLH1*, thereby enhancing the possibility of the causative mutation being different in various ethnicities.

The prognosis of LUS cancer has been examined in two small-scale studies (26,28). Patients with Lynch syndrome and concomitant colorectal cancer usually have a good prognosis (34,35). The prognosis of endometrial cancer in patients with Lynch syndrome has not been established, the findings for colorectal cancer, however, suggest that the prognosis of LUS cancer may be good for patients with Lynch syndrome. Notably, a comparative study on germline MMR mutation- or *hMLH1* hypermethylation-induced endometrial cancer showed an older onset age with fewer grade 1 and more grade 3 cases in the hypermethylation group (36). Therefore, the possible association of *hMLH1* hypermethylation with LUS cancer demonstrated in this study suggests a worse prognosis for LUS cancer associated with Lynch syndrome. LUS cancer is rare and relatively few cases have been described, thus, additional large-scale studies are required to establish the characteristics of this disease.

## Figures and Tables

**Table I:** Clinical characteristics of LUS and non-LUS tumors.

	LUS tumor		Non-LUS tumor		P-value
Median age (years)	44.4 (34.2–54.6)^a^		59.48 (55.8–63.1)^a^		0.001
Median BMI (g/m^2^)	18.5 (17.0–19.9)^a^		22.7 (21.2–24.1)^a^		0.002
Median parity	1.1 (0.3–1.9)^a^		1.5 (1.1–1.9)^a^		0.267

	No. of patients	%	No. of patients	%	P-value

Cesarean section					
No	7	77.7	26	96.2	0.082
Yes	2	22.2	1	3.7	
Infertility					
No	8	88.8	26	96.2	0.401
Yes	1	11.1	1	3.7	
Diabetes					
No	8	88.8	23	85.1	0.781
Yes	1	11.1	4	14.8	

a 95% confidence interval.

**Table II:** Pathologic characteristics of LUS and non-LUS tumors.

	LUS tumor		Non-LUS tumor		
			
	No. (N=9)	%	No. (N=27)	%	P-value
Histology					0.728
Endometrioid	8	88.8	25	92.5	
Non-endometrioid	1	11.1	2	7.4	
Histological grade					0.832
1	5	55.5	13	48.1	
2	3	33.3	7	25	
3	1	11.1	6	22.2	
Vascular invasion					0.841
Positive	6	66.6	17	62.9	
Negative	3	33.3	10	37	
Myometrial invasion					0.401
negative	1	11.1	1	3.7	
<1/3	3	33.3	12	44.4	
1/3-1/2	3	33.3	4	14.8	
>1/2	2	22.2	10	37	
Lymph node metastasis					0.579
Negative	7	77.7	19	73	
Positive	2	22.2	7	26.9	
Stage					0.665
I/II	6	66.6	18	66.6	
III/IV	3	33.3	9	33.3	

**Table III:** Microsatellite status and immunohistochemical (IHC) characteristics of LUS and non-LUS tumors.

	LUS tumor		Non-LUS tumor		
			
	No.	%	No.	%	P-value
MSI					0.39
MSI-H	2	22.2	7	25.9	
MSI-L	1	11.1	0	0	
MSS	6	66.6	20	74	
IHC					
Loss of hMLH1 expression	4	44.4	4	14.8	0.66
Loss of hMSH2 expression	0	0	4	14.8	0.66
Loss of hMSH6 expression	1	11.1	11	40.7	0.41

**Table IV:** IHC and MSI and methylation status of LUS patients.

	IHC						
					
Pt	hMLH1	hMSH2	hMSH6	MSI	h*MLH1* hypermethylation	Age	Amsterdam II criteria
1	−	+	+	MSS	−	62	Negative
2	−	+	−	MSI-H	+	35	Negative
3	+	+	+	MSS	−	46	Negative
4	+	+	+	MSS	−	39	Negative
5	+	+	+	MSS	−	26	Negative
6	+	+	+	MSS	−	69	Negative
7	+	+	+	MSS	−	41	Negative
8	−	+	+	MSI-L	+	40	Negative
9	−	+	+	MSI-H	−	42	Positive

IHC, immunohistochemistry; MSI, microsatellite instability; MSS, microsatellite stable.

## References

[1] Siegel R, Naishadham D, Jemal A (2012). Cancer statistics. CA Cancer J Clin.

[2] Forouzanfar MH, Foreman KJ, Delossantos AM (2011). Breast and cervical cancer in 187 countries between 1980 and 2010: a systematic analysis. Lancet.

[3] Saso S, Chatterjee J, Georgiou E, Ditri AM, Smith JR, Ghaem-Maghami S (2011). Endometrial cancer. BMJ.

[4] Brinton LA, Berman ML, Mortel R (1992). Reproductive, menstrual, and medical risk factors for endometrial cancer: results from a case-control study. Am J Obstet Gynecol.

[5] Gruber SB, Thompson WD (1996). A population-based study of endometrial cancer and familial risk in younger women. Cancer and Steroid Hormone Study Group. Cancer Epidemiol Biomarkers Prev.

[6] Lynch HT, de la Chapelle A (2003). Hereditary colorectal cancer. N Engl J Med.

[7] Peltomäki P (2003). Role of DNA mismatch repair defects in the pathogenesis of human cancer. J Clin Oncol.

[8] Dunlop MG, Farrington SM, Carothers AD (1997). Cancer risk associated with germline DNA mismatch repair gene mutations. Hum Mol Genet.

[9] Aarnio M, Sankila R, Pukkala E (1999). Cancer risk in mutation carriers of DNA-mismatch-repair genes. Int J Cancer.

[10] Lu KH, Dinh M, Kohlmann W (2005). Gynecologic cancer as a ‘sentinel cancer’ for women with hereditary nonpolyposis colorectal cancer syndrome. Obstet Gynecol.

[11] Vasen HF, Watson P, Mecklin JP, Lynch HT (1999). New clinical criteria for hereditary nonpolyposis colorectal cancer (HNPCC, Lynch syndrome) proposed by the International Collaborative Group on HNPCC. Gastroenterology.

[12] Vasen HF, Mecklin JP, Khan PM, Lynch HT (1991). The International Collaborative Group on Hereditary Non-Polyposis Colorectal Cancer (ICG-HNPCC). Dis Colon Rectum.

[13] Banno K, Susumu N, Hirao T (2003). Identification of germline MSH2 gene mutations in endometrial cancer not fulfilling the new clinical criteria for hereditary nonpolyposis colorectal cancer. Cancer Genet Cytogenet.

[14] Hendriks YMC, Wagner A, Morreau H (2004). Cancer risk in hereditary nonpolyposis colorectal cancer due to MSH6 mutations: impact on counseling and surveillance. Gastroenterology.

[15] Berends MJW, Wu Y, Sijmons RH (2003). Toward new strategies to select young endometrial cancer patients for mismatch repair gene mutation analysis. J Clin Oncol.

[16] Lu KH, Schorge JO, Rodabaugh KJ (2007). Prospective determination of prevalence of lynch syndrome in young women with endometrial cancer. J Clin Oncol.

[17] Millar AL, Pal T, Madlensky L (1999). Mismatch repair gene defects contribute to the genetic basis of double primary cancers of the colorectum and endometrium. Hum Mol Genet.

[18] Boland CR, Thibodeau SN, Hamilton SR (1998). A National Cancer Institute Workshop on Microsatellite Instability for cancer detection and familial predisposition: development of international criteria for the determination of microsatellite instability in colorectal cancer. Cancer Res.

[19] Simpkins SB, Bocker T, Swisher EM (1999). MLH1 promoter methylation and gene silencing is the primary cause of microsatellite instability in sporadic endometrial cancers. Hum Mol Genet.

[20] Goodfellow PJ, Buttin BM, Herzog TJ (2003). Prevalence of defective DNA mismatch repair and MSH6 mutation in an unselected series of endometrial cancers. Proc Natl Acad Sci USA.

[21] Hampel H, Frankel W, Panescu J (2006). Screening for Lynch syndrome (hereditary nonpolyposis colorectal cancer) among endometrial cancer patients. Cancer Res.

[22] Umar A, Boland CR, Terdiman JP (2004). Revised Bethesda Guidelines for hereditary nonpolyposis colorectal cancer (Lynch syndrome) and microsatellite instability. J Natl Cancer Inst.

[23] Lancaster JM, Powell CB, Kauff ND (2007). Society of Gynecologic Oncologists Education Committee statement on risk assessment for inherited gynecologic cancer predispositions. Gynecol Oncol.

[24] Westin SN, Lacour RA, Urbauer DL (2008). Carcinoma of the lower uterine segment: a newly described association with Lynch syndrome. J Clin Oncol.

[25] Sternberg S (1997). Normal histology of the uterus and fallopian tubes. Histology for Pathologists.

[26] Jacques SM, Qureshi F, Ramirez NC, Malviya VK, Lawrence WD (1997). Tumors of the uterine isthmus: clinicopathologic features and immunohistochemical characterization of p53 expression and hormone receptors. Int J Gynecol Pathol.

[27] Hachisuga T, Kaku T, Enjoji M (1989). Carcinoma of the lower uterine segment. Clinicopathologic analysis of 12 cases. Int J Gynecol Pathol.

[28] Hachisuga T, Fukuda K, Iwasaka T, Hirakawa T, Kawarabayashi T, Tsuneyoshi M (2001). Endometrioid adenocarcinomas of the uterine corpus in women younger than 50 years of age can be divided into two distinct clinical and pathologic entities based on anatomic location. Cancer.

[29] Masuda K, Banno K, Yanokura M (2011). Carcinoma of the lower uterine segment (LUS): clinicopathological characteristics and association with Lynch syndrome. Curr Genomics.

[30] Calle EE, Rodriguez C, Walker-Thurmond K, Thun MJ (2003). Overweight, obesity, and mortality from cancer in a prospectively studied cohort of U.S. adults. N Engl J Med.

[31] Watanabe Y, Nakajima H, Nozaki K (2001). Clinicopathologic and immunohistochemical features and microsatellite status of endometrial cancer of the uterine isthmus. Int J Gynecol Pathol.

[32] Edelmann L, Edelmann W (2004). Loss of DNA mismatch repair function and cancer predisposition in the mouse: animal models for human hereditary nonpolyposis colorectal cancer. Am J Med Genet C Semin Med Genet.

[33] Ollikainen M, Abdel-Rahman WM, Moisio A-L (2005). Molecular analysis of familial endometrial carcinoma: a manifestation of hereditary nonpolyposis colorectal cancer or a separate syndrome?. J Clin Oncol.

[34] Watson P, Lin KM, Rodriguez-Bigas MA (1998). Colorectal carcinoma survival among hereditary nonpolyposis colorectal carcinoma family members. Cancer.

[35] Lanspa SJ, Lynch HT, Smyrk TC (1990). Colorectal adenomas in the Lynch syndromes. Results of a colonoscopy screening program. Gastroenterology.

[36] Broaddus RR, Lynch HT, Chen L-M (2006). Pathologic features of endometrial carcinoma associated with HNPCC: a comparison with sporadic endometrial carcinoma. Cancer.

